# Evaluation of Heavy Metal Removal from Wastewater in a Modified Packed Bed Biofilm Reactor

**DOI:** 10.1371/journal.pone.0155462

**Published:** 2016-05-17

**Authors:** Shohreh Azizi, Ilunga Kamika, Memory Tekere

**Affiliations:** Department of Environmental Sciences, School of Agriculture and Environmental Sciences, University of South Africa, P. O. Box 392, Florida, 1710, South Africa; Federal University of Rio de Janeiro, BRAZIL

## Abstract

For the effective application of a modified packed bed biofilm reactor (PBBR) in wastewater industrial practice, it is essential to distinguish the tolerance of the system for heavy metals removal. The industrial contamination of wastewater from various sources (e.g. Zn, Cu, Cd and Ni) was studied to assess the impacts on a PBBR. This biological system was examined by evaluating the tolerance of different strengths of composite heavy metals at the optimum hydraulic retention time (HRT) of 2 hours. The heavy metal content of the wastewater outlet stream was then compared to the source material. Different biomass concentrations in the reactor were assessed. The results show that the system can efficiently treat 20 (mg/l) concentrations of combined heavy metals at an optimum HRT condition (2 hours), while above this strength there should be a substantially negative impact on treatment efficiency. Average organic reduction, in terms of the chemical oxygen demand (COD) of the system, is reduced above the tolerance limits for heavy metals as mentioned above. The PBBR biological system, in the presence of high surface area carrier media and a high microbial population to the tune of 10 000 (mg/l), is capable of removing the industrial contamination in wastewater.

## Introduction

In recent times concerns have been raised about the contamination of the environment with heavy metals. The discharges of industrial wastes that contain heavy metals present a potential hazard to an aquatic environment [1–4]. The elimination of these heavy metals contaminants can be accomplished by applying various established techniques to treat industrial wastewater streams, including methodologies that either reduce or precipitate via chemical means, ion exchange, electro-chemical methods and reverse osmosis. However, all these may prove unsuccessful, especially for solutions with 1 to 100 (mg/l) of metal concentrations [5–8]. Heavy metals have been proven to be toxic to microorganisms when they exceed allowable concentration limits. High concentrations of heavy metals affect microbial activity in the system and impede biological wastewater process [4, 9].

Heavy metals biosorption by microbial biomass is a new cost-effective method that has been developed to remove heavy metals from wastewater [3, 10, 11]. Bacteria, algae, fungi and yeasts have been examined to determine whether they act as metal biosorbents as they have metal sequestering properties. The interaction of microbial substances with heavy metals reduces heavy metal ion concentrations in solution [8, 12]. The efficient removal of heavy metals from wastewater is dependent on a several factors, including sludge concentration, the solubility of metal ions, pH, the metallic species and its concentration, and wastewater pollution load [9, 13]. A PBBR is based on attached growth technology, creating a biofilm on the supporting media, thus the amount of biomass concentration is of paramount importance for removing heavy metals and organic substances from the system. The efficient removal of heavy metals from the PBBR system has been the focus of the biosorption process. Independent of the metabolic process, biosorption can bind heavy metallic species to live cells, an inert biomass or to a microbial extracellular polymer [14, 15]. The biological system is a multifaceted process and depends on many biological and physicochemical variables. It may also depend on the design and operation of a system [9, 12]. The diversity of the biofilm’s microbial flora is measurably greater than that of a typical activated sludge system [16, 17].

The most significant organisms in any biological attached growth treatment process are bacteria that are mainly responsible for degrading organic matter and toxic waste, specifically heavy metals. While biofilm systems are susceptible to heavy metal ions and compounds, the systems are also resilient and robust, and they have a large diversity of bacteria that may allow the system to tolerate or even degrade heavy metals at specific concentrations [18, 19]. This susceptibility of a biological system is affected mostly by the bacteria and the cellular damage caused by exposure to toxic conditions.

This research presents the results of an evaluation of the selective removal of heavy metals (Cd, Cu, Ni and Zn) via a modified PBBR. The aim was to assess the efficacy of a PBBR biological process that removes heavy metals from the system as well as the attached growth biological system simultaneously driving the removal of organic contaminants. The influence of the species form (i.e. dissolved versus particle) on metal separation at different stages of the treatment processes was also evaluated. The capability of the modified PBBR was assessed for heavy metal removal at different metal concentrations.

## Materials, Methods and Setups

### Pilot plant setup

**Fig 1:**
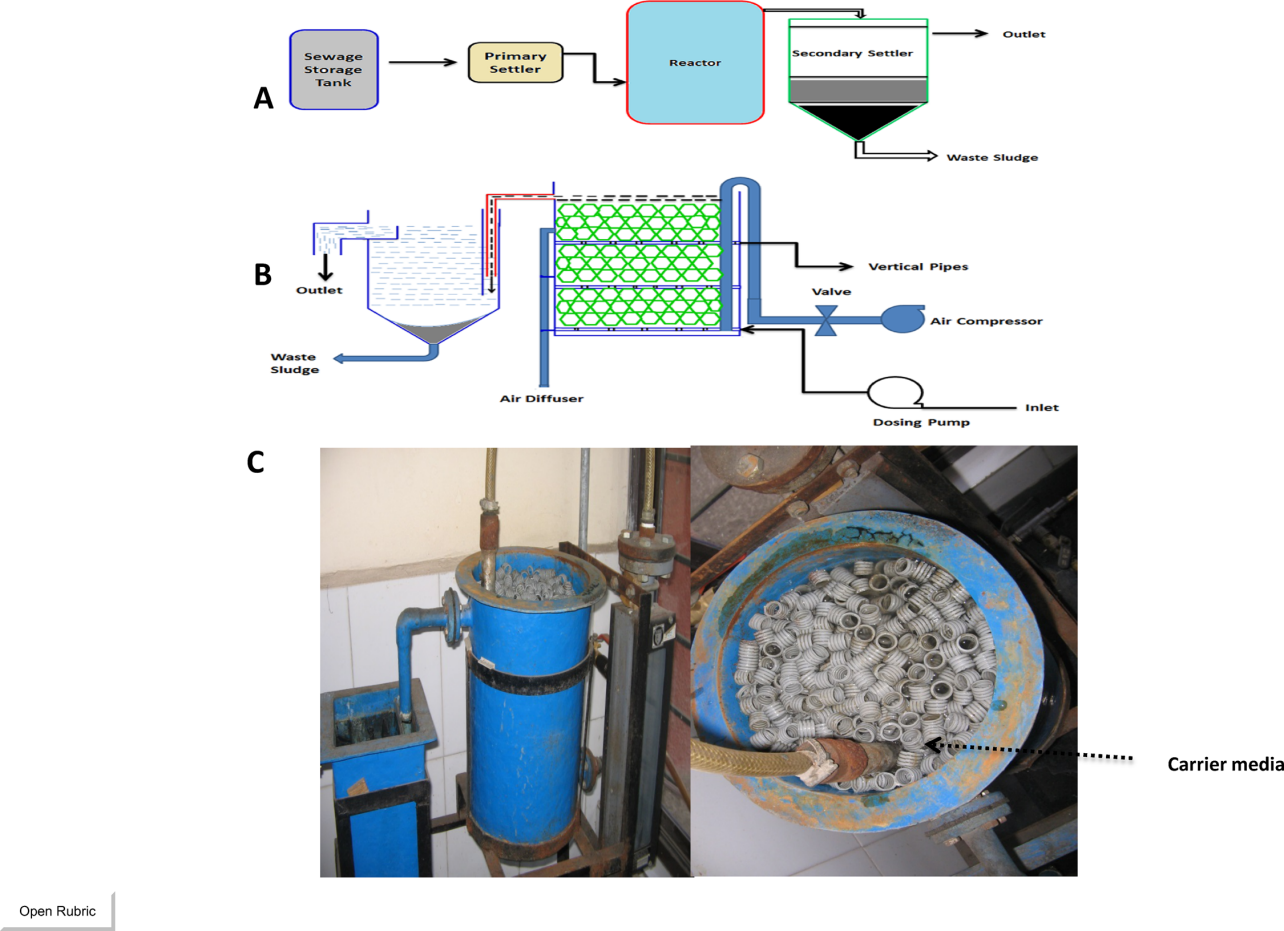
The schematic appearance of the PBBR (A), a detailed description of the reactor and secondary settler (B), and an image of laboratory scale system (C) [20]

A biological reactor similar to a previously reported PBBR system [20] was used to evaluate the tolerance of different strengths of composite heavy metals in the system. The laboratory unit is illustrated in **Fig 1.**

The key characteristic of the PBBR system is the arrangement of the fixed bed in the layered strata and the vertical pipe arrangement which ensures ease of effluent flow to increase the oxygen transfer in each layer. The void ratio of the reactor was calculated to be 92.18%. Controlled sewage was fed into the bottom of the reactor and sufficient up-flow velocity prevented clogging. The carrier media was submerged in effective volume of the reactor was approximately 10 litres (Fig 1). The combined heavy metals were added to the domestic wastewater inlet in the sewage storage tank. The reactor was filled with polypropylene (the carrier media) with a density of 0.95 g/cm^3^ and an active surface area of 350 m^2^/m^3^ consisting of outer grooves to prevent biofilm loss and promote growth of biofilm (Table 1).

**Table 1:** Details of Packed Bed Bioreactor and carrier media.

Reactor configuration		Carrier media feature	
Features	Details	Material	Polypropylene
Area of the reactor (m^2^)	0.035	Density (g/cm³)	0.95
Height of the reactor (mm)	320	Shape	Corrugated cylinder
Volume of the reactor (l)	11	Length (mm)	10
Void volume in presence of carrier media (l)	10	Specific surface area (m²/m³)	350
Settler volume (l)	2.68		

### Sampling and analysis

#### Experimental procedure

**Domestic wastewater characteristic:** The wastewater samples were collected daily from a local Mafikeng wastewater treatment plant to carry out an extensive experiment over 6 months. Mafikeng local municipality covers an area of 3698.44 km^2^; it has a population of 293 180 people and 69 397 households. The plant receives only domestic wastewater. The wastewater sample was transferred to a storage tank, as shown in Fig 1, and controlled sewage (the substrate) was continuously fed to the reactor. No specific permissions were required for the collection of wastewater samples as the plant is co-managed by the local government and the university. The present study did not involve any endangered or protected species. The responsible process controller assisted in collecting the wastewater sample.

**Metal solutions:** Composite metal concentrations were prepared by dissolving 2 mg/l, 5 mg/l, 7mg/l and 10 mg/l (at a time) of cadmium chloride hydrate (CdCl_2._H_2_O), zinc sulphate heptahydrate (ZnSO_4._7H_2_O), nickel sulphate hexahydrate (NiSO_4_.6H_2_O), and copper sulphate pentahydrate (CuSO_4_.5H_2_O) salts purchased from Sigma Aldrich (St Louis, Missouri, United States) in domestic wastewater to produce composite strengths of strengths of 8, 20, 28 and 40 mg/l respectively (**Table 2**).

**Table 2:** Variation of strength of composite heavy metals (Cd, Zn, Ni and Cu).

Composite heavy metals	Details
8	Contain 2 mg/l of each metal
20	Contain 5 mg/l of each metal
28	Contain 7 mg/l of each metal
40	Contain 10 mg/l of each metal

**Start-up and loading strategy:** Three litres of activated sludge was obtained from a returned sludge line of Mafikeng wastewater treatment plant. The activated sludge was added to the reactor to provide the initial microbial mass, after which 7 litres of domestic wastewater was added. During the first run, the reactor was filled with 60% (V/V) of carrier media. The hydraulic regime of the PBBR was slowly increased from 25% flow rate to 100% flow rate over a period of 25 days. During this time, the hydraulic retention time (HRT) was kept at 14 hours. It was established that the biomass on the carrier media was 6 500 mg/l in the 60 (V/V). The PBBR was examined in the presence of 2 mg/l of each heavy metal during the second run by adding carrier media till it reached 100 V/V of the reactor. The reactor was run for 8 days or till biomass concentration in the reactor reached 10 000 mg/l. The same concentration of heavy metals was examined and after comparisons of different carrier media, the carrier media in the reactor was optimised at 100 (V/V). A hydraulic retention time (HRT) of 2 hours (previously optimised in another study) [20] was also for this study. It should be mentioned that the bacterial mass of the activated sludge was determined as described by Sekar et al. [21]. The bacterial mass at the inlet and outlet was predominant (98.9%) with the following bacterial species: *Acinetobacter baumanni*, *Enterobacter gergoviae*, *Endwardsiella hoshinae*, *Klebsiella pheumoniae*, *Acinetobacter lwoffii*, *Moraxella lacunta*, *Escherichia coli*, *Pseudomonas putida*, *Acinetobacter haemolyticus*, *Enterobacter cloacae*, *Pseudomonas stutzeri*, *Aeromonas hydrophilia*, *Aeromonas sobria*, *Salmonella sp*, *Pseudomonas aeruginosa*, *Citrobacter koseri*, *Aeromonas salmonicida*. *koseri*, *Moraxella lacunta*; while in the carrier media, the following bacterial species was predominant (97.95%): *Acinetobacter haemolyticus*, *Acinetobacter lwoffii*, *Aeromonas sobria*, *Endwardsiella hoshinae*, *Citrobacter freundii*, *Klebsiella pheumoniae*, *Entrobacter gergoviae*, *Pseudomonas stutzeri*, *Serratia marcescens*, *Bacillus circulans*, *Chromobacter denitrificans*.

**Analysis:** The reactor was sampled daily at the inlet and outlet for the analytical determination of the chemical oxygen demand (COD), the biochemical oxygen demand (BOD), suspended solids (SS) and the biomass inside the carrier media. Analytical values were taken to be the mean of five replicates.

**Determination of biomass and suspended solids in the reactor:** The biomass content in the reactor was measured in terms of mixed liquor suspended solid (MLSS) and estimated by scraping off the surface of the carrier media in a known volume of wastewater. Then followed a suspended solid (SS) procedure according to standard methods (APHA_AWWA_WEF, 2005) [20, 22]. Briefly, a known volume of sample was filtered through a weighed standard glass fiber filter (Whatman 934-AH) and the residue on the filter was dried up to a constant weight at 103 to 105°C in an oven (CHF097). The increase in weight of the filter represents the total suspended solids.

**Determination of COD and BOD:** To test for COD concentration in the collected samples, the open reflux method was performed as reported by APHA_AWWA_WEF (2005). Briefly, 50 ml of each sample was pipetted and placed into a 500-ml refluxing flask with addition of 1 g HgSO4, several glass beads, and 5.0 mL sulfuric acid reagent. The mixture was well mixed to dissolve HgSO4 and cool while mixing to avoid possible loss of volatile materials. Furthermore, 25.00 ml of K_2_Cr_2_O_7_ solution, 70 ml of sulfuric acid, and 5.5 mg of Ag_2_SO_4_ were also added in the mixture. Solutions from the refluxing flask were well mixed and transferred in the vials to be heated at the reactor block for two hours at 150°C. After two hours, the vials are removed from the block to a cooling rack for about 15 minutes and COD were measured using a colorimeter. For BOD, the 5-day BOD standard method as reported by APHA_AWWA_WEF (2005) was used. The following method was based by measuring the difference in the oxygen concentration of the original sample and the sample when it has been incubated for 5 days at 20°C. The analytical values were the mean of five replicates. The performance evaluation for COD and BOD was based on effluent discharge norms specified by the local pollution control board.

**Determination of metal concentration:** The heavy metal concentration was determined using the atomic absorption spectrophotometer (AAS) (Model AA-6300 SHIMADZU). The determination of heavy metals in their particulate and dissolved forms was done according to a procedure reported by Karvelas et al [23]. Centrifugation of the inlet and outlet reactor samples was completed during a 30 minute at 4 000 (rpm) and at 4°C. A nitrate cellulose filter with a diameter of 0.45 μm was then used to filter the content prior to the completion of a digestion procedure where nitric acid was used. These samples were represented as the dissolved metals in a wastewater stream. The samples that remained were digested with aqua regia and were considered to be the total heavy metal content.

#### Quality control/quality assurance metals

Several quality control methods were used in order to obtain reliable data. The accuracy of the result in terms of the linearity of the calibration curve, the repeatability of results, the presence of any target metals in the blank solution and the standard deviation between the 5 replicates were assessed. To quantify metal contents from samples, external standards with calibration levels ranging from 2 to 10 mg/l for each metal were used. During the experiment, atomic absorption (AAS) used to quantify the targeted metals revealed good linearity with r ≥ 0.99. The precision of the analysis was tested on the instrument by analysing 5 replicate samples with SD ranging between 1.32 to 3.72, and the RSD ≥ 10. Hence, from the SD values it is possible to observe high repeatability of the analysis results. To ensure the accuracy of the results, the concentration of targeted metals in the blank solution (Ni 10 μg/l, Zn 4 μg/l, Cd6 μg/l, and Cu 2 μg/l) was subtracted from each analysed sample concentration. In addition, the limits of detection (LOD) of AAS was determined using linear regression and appeared to be 6 μg/l for Ni, 0.8 μg/l for Cd, 1.5 μg/l for Zn and 1.5 μg/l for Cu.

## Results and Discussion

The potential of unconventional compact wastewater treatment for heavy metal removal is considered for industrial activities in developed countries as an effluent standard for heavy metal removal where the standards have been tightened due to strict regulation and legislation.

### Comparative heavy metal removal in different percentages of carrier media in PBBR

The system was examined for two different quantities of MLSS concentration. The results showed that a decrease in fill ratio percentages led to a decrease in the efficiency of heavy metal reduction due to the reduced availability of surface area for microbial biofilm formation. The lower fill ratio carrier media percentage provided the least amount of biomass. It is theorised that heavy metal removal is dependent on the amount of available biomass; when there is an abundant amount of biomass there is a greater amount of removal [24, 25]. According to the results displayed in **Table 3**, the increase in the removal of heavy metals at higher MLSS concentrations was greater when compared to lower MLSS values. The system was developed with a higher biomass concentration of approximately 10 000 (mg/l) and experiments were conducted in this configuration.

**Table 3:** Heavy metal reduction for different fill ratio percentages of media.

Metal	Conc. (mg/l)	Percentage metal removal efficiency in fill ratio of media 100 (V/V)	Percentage metal removal in fill ratio of media 60 (V/V)
		Mean ± SD	Mean ± SD
Cu	2	85.28 ± 1.32	54.43 ± 3.65
Ni	2	76.32 ± 3.12	48.62 ± 2.98
Cd	2	71.01 ± 3.29	44.23 ± 2.74
Zn	2	80.43 ± 2.86	53.62 ± 3.72

*The values represent the mean of 5 replicates.

### Evaluations of heavy metal reduction in effluent wastewater in PBBR

The difference between heavy metal composition in the inlet and the outlet of the reactor was ascertained for heavy metal reduction via the PBBR process. It is possible that heavy metal reduction takes places mainly in two stages of biological treatment: the primary stage (when some metals are absorbed by particles) and the secondary stage (biosorption metal removal) [9, 14]. The PBBR was mainly designed for effective organic and nutrient removal using a biofilm system. As such the removal of heavy metals via the PBBR could prove to be an additional advantage.

**Fig 2:**
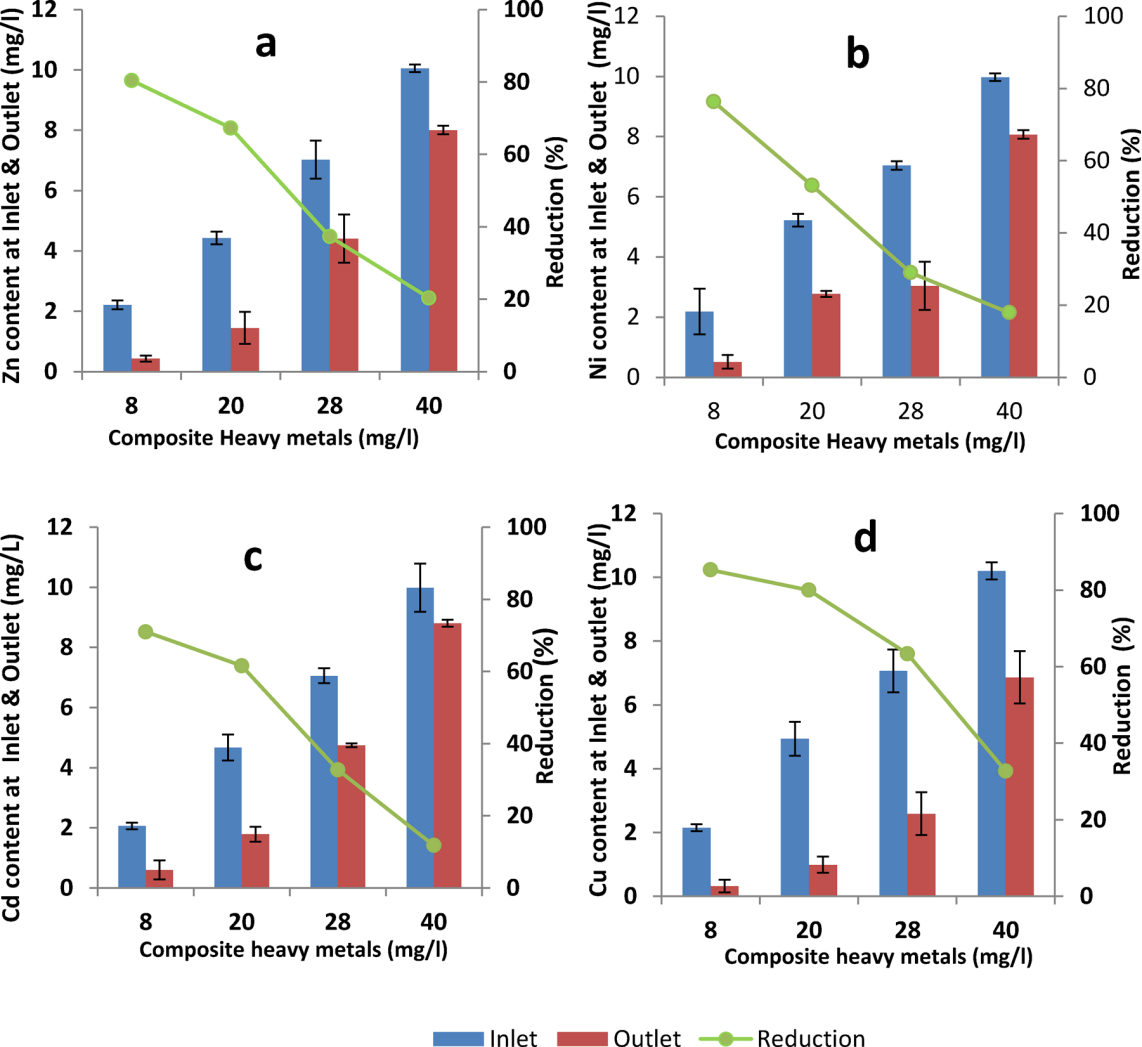
Heavy metal removal for different inlet and outlet concentrations. (a) Zn, (b) Ni, (c) Cd, (d) Cu.

Fig 2A, 2B, 2C and 2D displays the data gathered from the study. The heavy metals (Cu, Ni, Cd, and Zn) can be removed at the optimum HRT of 2 hours and the system was optimised by loading different heavy metal concentrations (8, 20, 28 and 40 mg/l) (Table 2). Heavy metal reduction at the outlet (with a concentration of 8 mg/l in the inlet, consisting of 2 mg/l of each metal) was higher than 71% (Fig 2A–2D).The higher removal efficiency occurred in the PBBR system when the heavy metal contents in the inlet were at 8 mg/l and 20 mg/l. Above these the removal efficiency was reduced. Other researchers report that biological processes are more effective to remove heavy metals at lower concentrations and also when more than one metallic ion are present in solution. At higher concentrations microorganisms are stressed, thus diminishing their action towards the metals [13, 26, 27]. Heavy metal removal efficiency is dictated by the influence of microorganisms. This becomes apparent at lower heavy metal concentrations due to stimulated microbial activity. Fig 2A–2D presents the following observed phenomena: by increasing the composite heavy metals concentration, especially at higher concentrations (i.e. >20 (mg/l)), the decrease in removal for the outlet stream could be due to the decreased activity of microorganisms in the system; and high concentrations can stress the microorganisms, thus reducing their action towards the metals [13, 28]. Stasinakis and Thomaidis [29] report that microbial respiration rate would be inhibited at higher concentrations of heavy metals in a biological system, thus causing a change in the microbial structure and adversely affecting the treatment process. The results indicated that the system was influenced by the starting inlet concentration, and removal efficiencies at low concentrations were greater than those in high concentrations [30]. Literature reports that the efficiency for removal increases at higher inlet metal concentrations (>0.5 (mg/l)). Nevertheless, it must be noted that efficient metal removal depends on the initial inlet metal concentration, process conditions, and the physical, chemical and biological variables of the wastewater treatment unit [14, 27, 30].

The reduction in the concentration of Cu (**Fig 2D**) for composite heavy metals to 8 and 40 mg/l in the outlet (85.28% and 32.69%, respectively) that was obtained was greater than that of other heavy metals. This finding resonates with reported findings in other studies which assessed heavy metal removal from the outlet streams of biological systems, in which the removal efficiency was highest for Cu [14, 30]. Microorganisms were not impacted negatively and maintained their functionality upon assimilating elemental Cu while in a biological treatment system [30]. Lester and John [27] report that the biomass found in the secondary treatment stage may have a fixed demand for this element [25]. A considerable reduction in the amount of heavy metals, ranging from 30% to 98%, was witnessed in a biological system [9, 29]. It is well documented that this phenomenon depends on the interaction between available active sites on biomass, the heavy metal species in the system, the composition of other wastewater components, and the biological operating system itself [14, 31]. The outlet removal efficiency for Cd concentration (**Fig 2C**) in composite heavy metals at 8 and 40 mg/l was 71.01% and 11.78%, respectively. This was observed to be less than that for other heavy metals studied. This can be ascribed to the elemental form of Cd itself, as well as the treatment system conditions [30]. Ajmal *et al* [32] report that inlet concentrations of Cd cannot be significantly reduced in biological wastewater treatment if the hydraulic retention time is less than 8 hours. The dominant species in the stream is Cd^2+^, classified as a weak acid with both a low oxidation state and electro-negativity value [33]. Ong et al [34] indicate that the maximum adsorption capacity of Cu, Ni and Cd ions onto activated sludge followed the order Cu > Ni > Cd, which was confirmed this research as follows: Cu > Zn > Ni > Cd.

However, some decreases in heavy metal content are achievable by combining multiple processes such as active bacterial uptake, cellular adsorption via bacteria, extracellular polymeric complexation as well as the containment of colloidal precipitant during flocculent formation [27].

### Organic matter removal at different strengths of composite heavy metals in the PBBR

**Fig 3:**
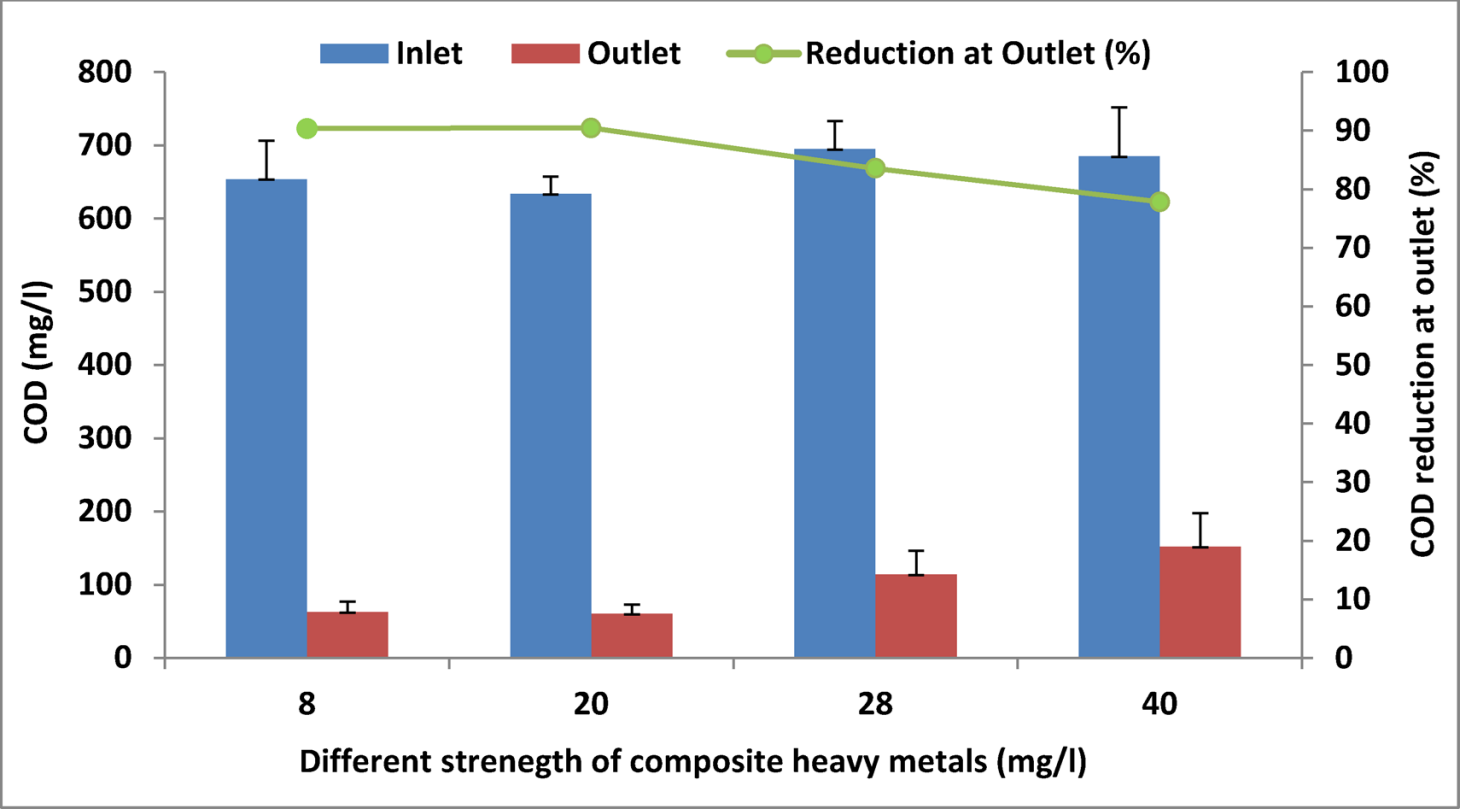
Average COD values at different composite heavy metal concentrations.

The application of microorganisms in the removal of heavy metals from wastewater has been effective and widely used [35]. Heavy metals at high concentrations are toxic to microorganisms, which may negatively affect the functioning of biological treatment process, especially removing organic matter [36]. In this study, organic matter removal rates with reference to COD and BOD concentration were evaluated to assess the performance of PBBR in the presence of different concentrations of heavy metals **(Table 4, Fig 3)**. The heavy metal concentrations in a PBBR based on attached system had no toxic effect up to a concentration of 20 mg/l. This concentration of heavy metals did not impact the treatment efficiency negatively, and the outlet COD and BOD concentrations were lower than 100 and 30 mg/l. However, the removal of organic matter decreased above this concentration level, indicating that heavy metal concentration above 20 mg/l would have a toxic effect on bacteria in the system.

**Table 4:** Experimental conditions at different hydraulic loadings.

Concentration of composite heavy metals	Initial BOD (mg/l)	BOD removed (mg/l)	Final BOD (mg/l)	Reduction at outlet (%)
8	281.23 ± 8.02	253.21 ± 7.42	27.22 ± 1.08	90.32
20	274.34 ± 10.31	250.98 ± 6.98	26.66 ± 1.41	90.28
28	278.98 ± 9.41	242.96 ± 9.10	33.75 ± 1.61	87.9
40	284.46 ± 8.89	233.65 ± 14.42	50.8 ± 1.59	82.14

The tolerance of the activated sludge process in the presence of the metals cadmium and zinc has been studied by Oviedo et al [37] to determine the toxic effect of metals on the system. Zinc has been observed to have some toxic effect on the system at concentrations up to 3 mg/l, while the inhibitory concentrations for Cd and Cu have been found to be 0.31 mg/l and 10 mg/l respectively [37, 38]. According to Hartmann et al [39] the toxic effects of metals on the activated sludge process were studied and the findings showed that the resistance of activated sludge from various wastewater treatment plants may vary because of differences in microbial community composition. There is a dearth of information on the effect of heavy metal concentrations on organic matter removal in attached biomass systems. In attached biomass systems, heavy metals ions at certain concentrations attack the slimy layer of the carrier media in the bioreactor, thereby inflicting damage on bacterial structures or essential bacterial activities. The subsequent damage created impacts the efficiency of organic matter removal in the system [19, 29].

### Heavy metal characterisation: the distribution of dissolved versus particulate forms

**Fig 4:**
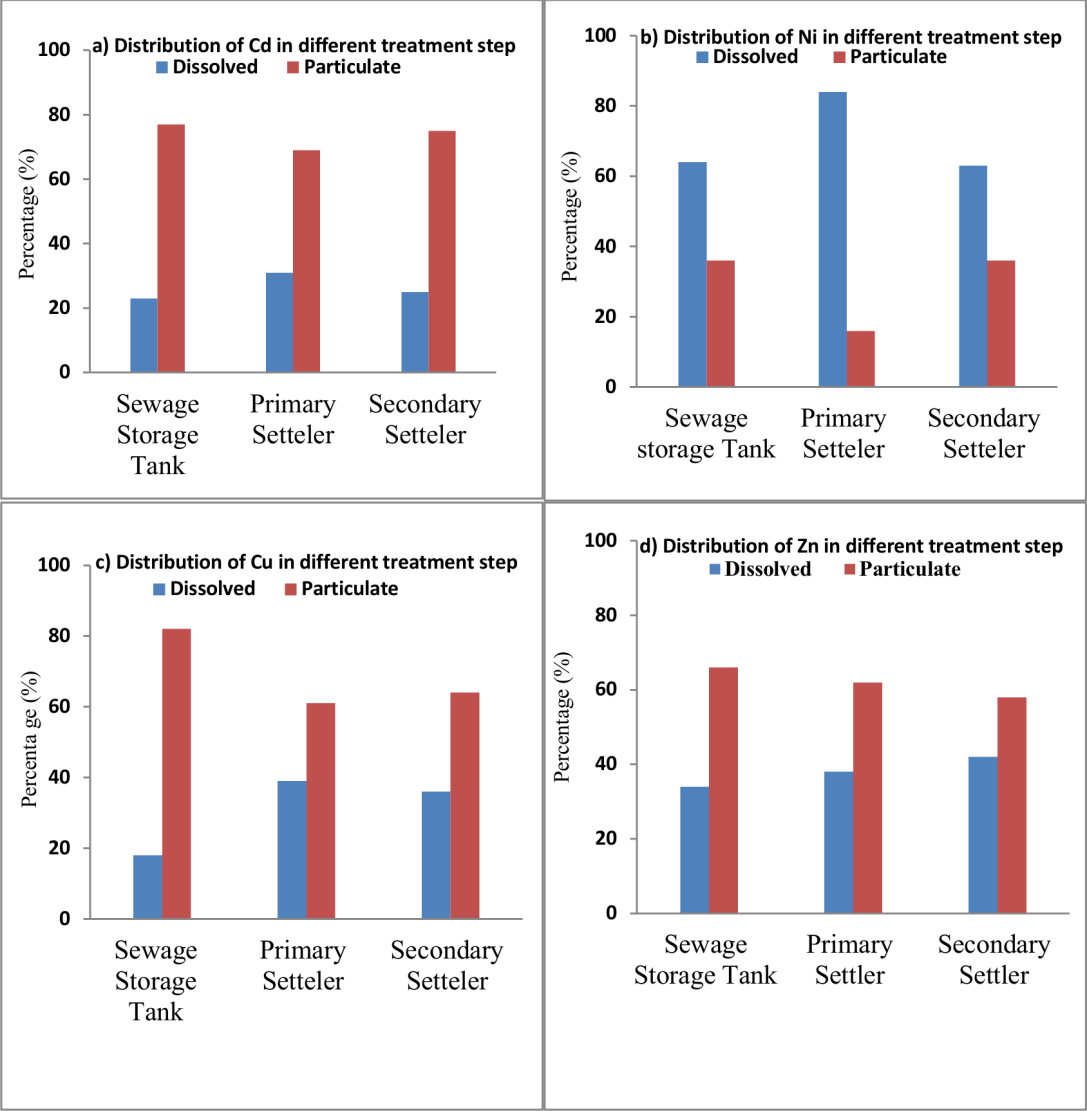
Heavy metal distribution in particulate and dissolved forms in different stages in the PBBR system. **(a)** Distribution of Cd in different treatment stages **(b)** Distribution of Ni in different treatment stages. (**c)** Distribution of Cu in different treatment stages **(d)** Distribution of Zn in different treatment stages.

Eight milligrams per litre (8 mg/l) of composite heavy metal consisting of 2 mg/l of each of the species (Ni, Cd, Cu, and Zn) were investigated at the different stages of the PBBR treatment system to determine the heavy metal distribution in both the particulate and the dissolved forms. These selected elements are of major interest due to their inherent toxic nature and the duration for which they linger in an ecosystem [40]. **Fig 4** shows the dissolved and particulate metal forms distribution for the PBBR system. Karvelas et al [23] describes the occurrence of small transformations in the phase distributions of individual metals during biological treatment process that cause slight increases in the amounts of the dissolved form of some metals after each treatment stage.

Primary sedimentation was observed to affect the distribution of Zn greatly, while biological processes and secondary sedimentation affected mostly the distribution of Cu, Cd and Ni [41]. This study also indicates that during different stages of treatment in the PBBR system, there are slight changes in the distribution of selected metals. Findings of a research by Chanpiwat et al 2010 also confirms that wastewater treatment appears to affect only slightly the phase distribution of metals such as Cu, Ni, Cr, Pb, Cd and Zn [30]. The reaction of these metals is also attributed to their specific chemical forms; each has its own unique water solubility value during the various condition levels in the treatment process [23, 42].

**Fig 4(C)** shows that in the sewage storage tank where heavy metals and wastewater are mixed, the dissolved form of Ni (64%) can be found during this stage. This percentage improves to 84% in the primary settler. The majority of Ni measured is in the dissolved form, which might be due to the high mobility of this element [23]. In this study, Cd, Cu and Zn are mostly associated with the particulate form (58 to 82%). As such, the particulate form of selected metals in the secondary settler can be ordered as follows: Cd > Cu > Zn > Ni. These results indicate that the PBBR treatment process is mainly affected by the distribution of Cu and Cd. It is less affected by the distribution of Ni and Zn, where increases in the dissolved phase were mainly exhibited in Ni (the highest at 84%).

## Conclusion

In this study, an effort has been made to remove heavy metals at different loading concentrations using a PBBR biological system. The conclusion of this study is that heavy metal removal efficiency at the outlet occurs at an optimum HRT of 2 hours. Furthermore, Cu and Zn are more readily removed than Cd and Ni (Cu > Zn > Ni > Cd). Additionally, a tolerable limit of 20 mg/l for composite heavy metals was established for PBBR treatment systems operating at optimum conditions over 2 hours, and concentrations above 20 (mg/l) were noted to affected adversely affect treatment efficiency. The distribution of heavy metals in both their particulate and dissolved phases during different stages of wastewater treatment shows that the particulate form of selected metals in secondary settler follows this order: Cd > Cu > Zn > Ni. It is therefore concluded that industrial wastewater effluents can benefit from the investigated PBBR, which is capable of removing heavy metal contamination.
